# The Retrograde and Retroperitoneal Totally Laparoscopic Hysterectomy for Endometrial Cancer

**DOI:** 10.1155/2012/263850

**Published:** 2012-07-13

**Authors:** Eugenio Volpi, Luca Bernardini, Anna Maria Ferrero

**Affiliations:** ^1^Department of Obstetrics and Gynecology, Saint Andrew Hospital Asl 5, La Spezia, Italy; ^2^Dipartimento Materno-Infantile, Ospedale Sant' Andrea, Asl 5, Via Veneto 134, 19100 La Spezia, Italy; ^3^Department of Gynecologic Oncology, University of Turin, Turin, Italy

## Abstract

*Introduction*. We retrospectively report our experience with the utilization of an original procedure for total laparoscopic hysterectomy based on completely retrograde and retroperitoneal technique for surgical staging and treatment of the endometrial cancer. The surgical, financial, and oncological advantages are here discussed. *Methods*. The technique used here has been based on a combination of a retroperitoneal approach with a retrograde and lateral dissection of the bladder and retrograde culdotomy with variable resection of parametrium. No disposable instruments and no uterine manipulator were utilized. *Results*. Intraoperative and postoperative complications were observed in 10% of the cases overall. Operative time length and mean haemoglobin drop value results were 129 min and 125 mL, respectively. Most patients were dismissed on days 3–5 from the hospital. Seventy-eight percent of the patients were alive with no evidence of disease at mean followup of 49 months. *Conclusions*. Our original laparoscopic technique is based on a retroperitoneal approach in order to rapidly control main uterine vessels coagulation, constantly check the ureter, and eventually decide type and site of lymph nodes removal. This procedure has important cost saving implications and the avoidance of uterine manipulator is of matter in case such as these of uterine malignancy.

## 1. Introduction

In the last decade, laparoscopy as well as robotics have been increasingly applied with success to patients with gynaecological malignancies, including endometrial cancer [1]. Laparoscopic surgical staging often used in conjunction with a vaginal hysterectomy provides an alternative therapeutic approach to the standard abdominal laparotomic staging of endometrial cancer [2]. When compared to laparotomy, the laparoscopic approach is indeed associated with a faster return to normal activity and reduced intra- and postoperative morbidity [3, 4]. More recently, as shown by an increasing number of prospective and randomized studies, total laparoscopic hysterectomy has become a standardized way for proper surgical treatment and staging of early endometrial cancer [5–12]. The totally laparoscopic approach has some advantages over the laparoscopy-assisted procedure, including the avoidance of losing time to shift from one operative field to another, a direct visualization of the vaginal cuff resection margins and enhanced removal of the uterus and lymph nodes in case of enlarged uteri or narrow vagina. This is not only our own opinion but also the opinion of others [4]. In addition, the average cost for laparoscopic hysterectomy and staging favourably compares with both laparotomy and robotics [13]. On the other side, the utilization of the uterine manipulator which is essential for all laparoscopic hysterectomies is of concern in case of uterine malignancies [14, 15]. In fact, in these cases, the fear for a peritoneal and systemic spread of tumor cells is a reasonable preoccupation. 

In this paper we describe our experience using a retrograde and retroperitoneal hysterectomy for minimally invasive comprehensive surgical staging of the endometrial cancer. In our opinion the retrograde and retroperitoneal approach allows to get optimal and constant protection of the ureter, faster control of unexpected intraoperative hemorrhages and better modulation of radicality.To this end we have adopted a combination of the retroperitoneal laparoscopic approach as originally described by Köhler et al. [16] and Roman et al. [17] with that of retrograde culdotomy reported long time ago by Delle Piane (1967) [18], Hudson and Chir (1968) [19], Robert [20], and more recently by Bristow et al. [21]. At our department, this surgical approach is routinely chosen for most of all the laparoscopies regardless of the malignancy. Always not disposable instruments nor uterine manipulator are employed. 

## 2. Materials and Methods

### 2.1. Patients

From January 2002 to December 2011, all patients with diagnosis of endometrial cancer were treated by the same team of gynecologic oncologists operators at two departmental hospitals: Turin and La Spezia. The analysis was based on the data of 95 patients. Data regarding patient characteristics and intraoperative details were elicited from an oncologic database developed for retrospective review. The patient characteristics retrieved were age, body mass index, concomitant diseases, previous surgeries, stage of disease according to the 2009 International Federation of Gynecology and Obstetrics [22], histopathologic subtype, and tumor grade. Intraoperative parameters included blood loss, perioperative blood transfusions, operative time, and number of pelvic lymph nodes removed. Postoperative parameters evaluated short-and long-term complications, postoperative adjuvant therapy (radio and chemotherapy), length of hospitalization, median followup duration, recurrence, and disease-free interval. Patients were not considered candidates for the laparoscopic approach in case of metastases beyond the uterus. Neither high body weight nor previous abdominal surgery was considered a contraindication for the laparoscopic approach. Pelvic and lumbar-aortic lymphadenectomies have been performed in most cases based upon the surgical staging (after hysterectomy) or as first surgical step (before hysterectomy) when preoperative information about grading and histologic subtype (G2 or serous papillary or clear cell or undifferentiated) would has warranted it. Informed consent was obtained for all patients about risks of anesthesia, hysterectomy, laparoscopy, and risk of conversion to laparotomy. All patients underwent general anaesthesia and endotracheal intubation. The day of surgery, Cefazolin 2 gr was i.v. administered. Always, prophylactic anticoagulation therapy was given for ten days. 

### 2.2. Surgical Technique

Patients were positioned in a dorsal lithotomy with legs apart and semiflexed, and the arms tucked at the sides. The surgical table was kept in a low position and the monitor between the patient's legs, facing the two surgeons to facilitate an ergonomic working position. A simplified equipment of no disposable instruments was used including a scissor, two grasping forceps, a washing-aspiration cannulae, and a 3 mm bipolar coagulation forceps. Ligasure (ValleyLab, Boulder, CO, USA) was used only for radical hysterectomies. Never the uterine manipulator was utilized. 

 A gasless access to the peritoneum was obtained by grasping the skin at the umbilicus with 2 Backhaus forceps and strongly elevating it while a 2 cm depth and 1 cm long incision was blindly made inside the umbilicus at its deepest part. A 10 mm trocar was then gently inserted throughout the incision to hold the laparoscope with the camera. When indicated by the surgical history of the patient, a Veress needle was first inserted into the peritoneal cavity at the left upper abdominal quadrant (Palmer site) to obtain intraperitoneal gas distension. At this point, patient was put in Trendelenburg position and three 5 mm trocars placed in the lower abdomen under direct vision. Two of these trocars were placed laterally to the epigastric vessels at the level of the superior iliac spine while the third one was centrally sovrapubic. 

An incision was made where the broad ligament overlies the psoas muscle thus allowing to enter into the pararectal space. The peritoneum was opened parallel to the infundibulopelvic ligament above the crossing with the external iliac artery and along the umbilical artery (which can be tracked upwards along the abdominal wall). An avascularized space of areolar tissue was developed by dissection between a medial leaflet of the broad ligament and the external and internal iliac vessel, taking care to dislocate the ureter on the medial leaflet and avoiding dissection laterally to the internal iliac artery. Following the course of the ureter by one side and the internal iliac artery by the other, the crossing of the uterine artery was encountered (generally 1-2 cm further back the origin of the superior bladder artery). The uterine artery was bipolarly coagulated over 1-2 cm distance. Often the uterine veins were grasped and coagulated altogether. 

When necessary a pelvic lymphadenectomy was performed either as first surgical step or following surgical staging. In any case it was performed bilaterally from the level of the aortic bifurcation along the external iliac vessels to the circumflex iliac vein. Internal iliac lymph nodes are then removed. The obturator lymph nodes were removed taking care to identify the obturator nerve. Para-aortic lymphadenectomy was not routinely performed unless suspicious pelvic lymph nodes or deep endometrial invasion or serous papillary or other abnormal histological types were present. Lymph nodes were removed altogether in one single endobag from the vagina at the end of the operation. The round ligament was only partially divided and the anterior leaf of the broad ligament utilized to prepare the paravesical space. A blunt dissection toward the pelvic floor between the superior bladder artery and the cervix was created on both sides. The vesicouterine peritoneal fold was left aside and retrograde dissection initiated from the sides of cervix. Cervical and vaginal uterine vessels were eventually identified, isolated, and coagulated. At the end of this time the transection of the round ligament was completed by dividing all the anterior leaf down to the vesicouterine peritoneal fold. This was finally mobilized from connections to the lower uterine segment. The infundibulopelvic ligaments were identified as high as possible out of the pelvis. While being grasped and elevated, an incision was bluntly made 1 cm beneath the ligament on the underlying peritoneum. This allowed to push away the ureter before coagulation. These ligaments were coagulated with bipolar over a 2 cm distance and were divided. 

Afterwards, the posterior margin of the peritoneum was superficially incised towards the posterior vaginal apex and rectovaginal septum. By creating the avascular space the medial portion of the sacrouterine ligament could be safely drawn away from the isthmic portion of the ureter. The apical part of the rectovaginal septum was then opened. During this step parametrial tissue containing the vascular pedicles was coagulated and variably dissected as a function of the radicality required. A third operator was then enrolled to expose, by means of ring forceps, the anterior vaginal vault which was therefore incised and opened by the first operator. A vaginal tampon was then used to stop gas loss and maintain the pneumoperitoneum. While the second operator was grasping the anterior margin of the vagina, the first operator executed the retrograde incision of the vagina (circular culdotomy). This was facilitated by pulling up the cervix and dissecting the vaginal mucosa at variable distance from the portio as indicated by need of radicality. During this final step the sacrouterine ligaments were coagulated and transected. The retrograde direction of the culdotomy proceeded parallel and 2-3 cm above the course of the ureter. The vagina was then sutured laparoscopically by using 14 cm 0 Quill SRS suture (Angiotech, Vancouver, BC, Canada). Closure started at one angle of the vaginal cuff and prosecuted in a running fashion with a final stich securing one uterosacral ligament to the other. Finally, the pelvis was washed and hemostasis assured.

## 3. Results 

The results are summarized in Tables 1, 2, and 3. Ninety-five patients affected by endometrial cancer at variable stage were consecutively referred to our attention. Clinical details of our patient population are given in Table 1. Conversion to laparotomy has never occurred. Endometrioid adenocarcinoma was the most common histological type found (70.5%). In the remaining 28 cases, rare histological subtypes associated with poor outcome were noted (29.1%). No invasion or less than one-half myometrial invasion was present only in 58.4% of the cases. A peritoneal cytology resulted positive in 12.6% of cases (12/95). In half of these cases the level of myometrial invasion resulted < of 50% (IA surgical stage). Cumulative prevalence of high grade (G2, G3) cases and advanced disease (IB, II, IIIA, IIIC stages) were 65.26% and 46.31%, respectively (Table 1). Always a total laparoscopic extrafascial hysterectomy coupled to bilateral annessiectomy was performed aside from 21 cases undergone radical hysterectomy (Table 2). This was decided based on preoperative information achieved throughout hysteroscopy or endocervical curettage or intraoperative surgical and pathological staging. Despite this, the surgical procedure here employed has basically remained unchanged for all the patients. Median operative time was 129.47 minutes (60–240). However, after excluding more complicated cases of fixed or enlarged uteri, the median operative time dropped significantly. In few instances a sudden bleeding in the pararectal space occurred but has always been promptly controlled by bipolar coagulation with no significant impact on the duration of the operation. In general, the blood loss was on average 125.15 mL (range 100–300). Mean length of patients hospitalization stay was 3.5 days (2–6) (Table 2). Three patients suffered short-term perioperative complications. One patient had an unintentional cystotomy that was recognized and repaired by laparoscopy while other two patients were found with ureteral injuries and undergone endoscopic positioning of a double J ureteral stent and one week hospitalization stay was needed. One patient required an intraoperative blood transfusion (1 U packed red blood cells) and another one received 2 U of packed red blood cells postoperatively. In 5 out of 96 cases, major postoperative complications occurred. This included one patient with advanced stage tumor who developed a vagina-enteral fistula (pelvic recurrence after 7 months and death), one with pelvic hematoma, one with pelvic abscess, one with septic lymphatic cyst, and another one with renal dilatation (Table 2). Adjuvant radio- or chemotherapy was administered in 29 patients (Table 3). A mean followup of 49.09 months (4–140) showed an important rate of 78.94% of patients with no evidence of disease (NED) (75/95). Thirteen had recurrences after a disease free interval of, on average, 15 months and all but one then died (12/13) (Table 3). These patients were suffering from advanced disease (abnormal histological subtypes and low grade). In our series, no port-site metastasis was ever observed. 

## 4. Discussion

In this study we have reported about the method applied at our department for total laparoscopic hysterectomy in case of endometrial cancer. As shown in Table 1 patients characteristics were typical for this type of disease. Most of them were overweight, had a medical history of previous abdominal laparotomies, and all of them had some additional important health problem. Abdominal surgery therefore would have exposed them to increased risk of complications. As suggested by Vergote et al. (2009) [23] these patients might have benefit in many instances of vaginal hysterectomy or, better, laparoscopy assisted vaginal hysterectomy. We have been employing a retroperitoneal laparoscopic hysterectomy since a long time and believe about important advantages of laparoscopy particularly in case of uterine cancer staging. We agree with Magrina, (2001) [24] that in selected patients and in the hands of gynecologic oncologists experienced in advanced laparoscopic techniques the laparoscopic approach provides major patient advantages and should be used whenever feasible. In our opinion it is not any longer the case to doubt whether is it safe to treat endometrial carcinoma endoscopically [23]. Concerns raised about recurrence and survival rates are questionable since they appear comparable to those obtained by laparotomy [12]. Despite the poor surgical quality, on average, of our study population, we have been able not to convert any patient to laparotomy. This is contrary to that reported by GOG studies [10, 11] in patients having similar age and BMI. This is even more remarkable considering the high percentage of patients at advanced stage of disease undergone laparoscopic treatment and staging in our study. As it refers to the intra- and postoperative complication and survival rates observed in our retrospective study they are in agreement with those generally reported by most part of the studies. In particular a 78% of patients with no evidence of disease after on average of 49 months followup is noteworthy. Again this is of relevance since our patient population was largely far from being ideal and not comparable to patients studied so far by other authors (early endometrial cancers). In our study the prevalence of bad histological subtypes and high grade tumors was indeed very high (21% and 65%, resp.). In our series only 20% were true early endometrial cancers making our study not comparable to other studies. Once again we can conclude that in terms of operative time, expected blood loss and duration of hospitalization our data confirm that laparoscopic hysterectomy compares favorably to laparotomic hysterectomy. The technique here used for hysterectomy has in particular the advantage of being particularly cost saving and safe in terms of potential spread of tumor cells since the uterine manipulator is avoided. The technique is reproducible and has been performed by the same operators respecting consistently any single surgical step each time. Amount of parametrium to be resected and extension of lymphadenectomy were modulated on the bases of either preoperative data (grading) or intraoperative factors (FIGO 2009 surgical staging). This was made possible because of the accurate retroperitoneal preparation of the uterine vessels at their origin and retrograde dissection of paravesical tissue as well as retrograde incision of the vagina. By adopting a standard retroperitoneal approach in all the cases, it was easier to modify grade of radicality and decide for a pelvic and/or para-aortic lymphadenectomy.

In conclusion, we confirm adequacy and cost effectiveness of laparoscopy for surgical staging and treatment of endometrial cancer. Specifically, our method of retrograde and retroperitoneal hysterectomy is particularly indicated and valuable in that it avoids the use of uterine manipulator and allows easy modulation of radicality. This last consideration is important since patients suitable for surgical treatment of endometrial cancer represent a quite heterogeneous population. 

## Figures and Tables

**Table 1:** Patients characteristics.

Patient's profile	
Number of cases	95
Age (years) mean (range)	63.46 (43–84)
BMI mean (range)	29.64 (20–46)
Other pathologies	
Hypertension	60
Diabetes	13
Thyroid	9
Other	13
Total (%)	95 (100%)
Previous surgeries	51
Histology	
Endometrioid	67
Adenosquamous	8
Serous-papillary	6
Villous-glandular	5
Undifferentiated	5
Mucinous	2
Carcinosquamous	1
Clear cell	1
Grading	
G1	33
G2	43
G3	19
Myometrial invasion	
No invasion	6
<50%	50
>50%	39
FIGO Staging	
IA	51
IB	26
II	3
IIIA	5
IIIC1	7
IIIC2	3
Positive washing cytology	12 (6/12 myom.invasion < 50%)

**Table 2:** Perioperative data.

	Number of cases
Hysterectomy + bilat. annessiectomy	95
*Extrafascial*	74
*Radical*	21
Operative time (min)	129.47 (60–240)
EBL (mL)^∗^	125.15 (100–300)
Pelvic lymphadenectomy	65
Mean number of pelvic lymph removed	10.25 (1–28)
Para-aortic lymphadenectomy and omentectomy	13
Mean number of hospitalization days	3.5 (2–5)
Intraoperative complications (2 blood transfusions, 3 ureteral injuries)	5.2% (5/95)
Postoperative complications (fistula, lymphocyst, ascess, renal dilatation, hematoma)	5.2% (5/95)

*EBL: bleeding loss.

**Table 3:** Adjuvant therapy, followup, and survival.

	Number of cases
Adjuvant radiotherapy	20
Adjuvant chemotherapy	9
No adjuvant therapy	66
Mean followup (months)	49.09 (4–140)
Lost	6
NED^∗^	75
ED^°^	1
Deaths	13
Recurrences	13 (12 deaths + 1 ED)
*Vaginal*	4
*Pelvic*	6
*Peritoneal*	3
Disease-free interval (months)	15 (7–34)

^∗^NED: no evidence of disease.

^°^ED: evidence of disease.
